# Mesenchymal stem/stromal cells from human pluripotent stem cell-derived brain organoid enhance the ex vivo expansion and maintenance of hematopoietic stem/progenitor cells

**DOI:** 10.1186/s13287-023-03624-w

**Published:** 2024-03-05

**Authors:** Ya Zhou, Xinping Cai, Xiuxiu Zhang, Yong Dong, Xu Pan, Mowen Lai, Yimeng Zhang, Yijin Chen, Xiaohong Li, Xia Li, Jiaxin Liu, Yonggang Zhang, Feng Ma

**Affiliations:** 1 Center for Stem Cell Research and Application, Institute of Blood Transfusion, Chinese Academy of Medical Sciences and Peking Union Medical College (CAMS & PUMC), Huacai Road 26, Chengdu, 610052 China; 2 State Key Laboratory of Experimental Hematology, National Clinical Research Center for Blood Diseases, Haihe Laboratory of Cell Ecosystem, Institute of Hematology and Blood Diseases Hospital, Chinese Academy of Medical Sciences and Peking Union Medical College(CAMS & PUMC), Tianjin, 300020 China; 3 Department of Immunology, School of Basic Medical Sciences, Chengdu Medical College, Chengdu, China

**Keywords:** MSCs, Human brain organoids, HSCs, Wnt signaling pathway, HSC transplantation

## Abstract

**Background:**

Mesenchymal stem/stromal cells (MSCs) are of great therapeutic value due to their role in maintaining the function of hematopoietic stem/progenitor cells (HSPCs). MSCs derived from human pluripotent stem cells represent an ideal alternative because of their unlimited supply. However, the role of MSCs with neural crest origin derived from HPSCs on the maintenance of HSPCs has not been reported.

**Methods:**

Flow cytometric analysis, RNA sequencing and differentiation ability were applied to detect the characteristics of stromal cells from 3D human brain organoids. Human umbilical cord blood CD34^+^ (UCB-CD34^+^) cells were cultured in different coculture conditions composed of stromal cells and umbilical cord MSCs (UC-MSCs) with or without a cytokine cocktail. The hematopoietic stroma capacity of stromal cells was tested in vitro with the LTC-IC assay and in vivo by cotransplantation of cord blood nucleated cells and stroma cells into immunodeficient mice. RNA and proteomic sequencing were used to detect the role of MSCs on HSPCs.

**Results:**

The stromal cells, derived from both H1-hESCs and human induced pluripotent stem cells forebrain organoids, were capable of differentiating into the classical mesenchymal-derived cells (osteoblasts, chondrocytes, and adipocytes). These cells expressed MSC markers, thus named pluripotent stem cell-derived MSCs (pMSCs). The pMSCs showed neural crest origin with CD271 expression in the early stage. When human UCB-CD34^+^ HSPCs were cocultured on UC-MSCs or pMSCs, the latter resulted in robust expansion of UCB-CD34^+^ HSPCs in long-term culture and efficient maintenance of their transplantability. Comparison by RNA sequencing indicated that coculture of human UCB-CD34^+^ HSPCs with pMSCs provided an improved microenvironment for HSC maintenance. The pMSCs highly expressed the Wnt signaling inhibitors SFRP1 and SFRP2, indicating that they may help to modulate the cell cycle to promote the maintenance of UCB-CD34^+^ HSPCs by antagonizing Wnt activation.

**Conclusions:**

A novel method for harvesting MSCs with neural crest origin from 3D human brain organoids under serum-free culture conditions was reported. We demonstrate that the pMSCs support human UCB-HSPC expansion in vitro in a long-term culture and the maintenance of their transplantable ability. RNA and proteomic sequencing indicated that pMSCs provided an improved microenvironment for HSC maintenance via mechanisms involving cell–cell contact and secreted factors and suppression of Wnt signaling. This represents a novel method for large-scale production of MSCs of neural crest origin and provides a potential approach for development of human hematopoietic stromal cell therapy for treatment of dyshematopoiesis.

**Supplementary Information:**

The online version contains supplementary material available at 10.1186/s13287-023-03624-w.

## Introduction

Hematopoietic stem cells (HSCs) reside in a heterogeneous microenvironment (or niche) in which they generate all functionally mature blood cells. The niches harboring HSCs are present in diverse tissues throughout ontogeny, beginning in the aorta-gonad-mesonephros (AGM) region, followed by the placenta, fetal liver, and spleen, before finally settling and persisting in the bone marrow (BM) after birth [1]. In parallel to the AGM region during the fetal stage, HSCs are also found in the mouse embryonic head [2]. It is widely accepted that MSCs represent a critical constituent of the HSC niche [3]. Adult mouse BM and embryonic stromal cell lines with mesenchymal phenotypes have been characterized for their function in supporting the growth, maintenance, and differentiation of mouse and human HSCs [4, 5]. The adult-type human MSCs, such as BM-derived MSCs (BM-MSCs), placenta-derived MSCs, and umbilical cord-derived MSCs (UC-MSCs), have the capacity to act as feeders to maintain the undifferentiated state of HSPCs, although efficacy varies depending on the cell source [6–8]. It has been demonstrated that MSCs derived from different tissues exhibit diversity in a range of biological characteristics and substantial heterogeneity [9, 10]. Furthermore, the number of MSCs harvested from a single-donor source is limited due to their restricted long-term proliferative capacity. Therefore, it remains critically challenging to produce homogeneous populations of MSCs with specific criteria for therapeutic applications.

Significant efforts have been made to identify alternative cell sources of MSCs, particularly by means of human pluripotent stem cells (hPSCs). Recent reports have shown that unlimited production of MSCs can be achieved using hPSC-derived mesoderm cells, neural crest cells (NCCs), and trophoblast-like intermediate cells [11–13]. These hPSC-derived MSCs represent a possible alternative source of therapeutic MSCs that can overcome the problems associated with standardized cell preparation and off-the-shelf production in clinical applications. The hPSC-derived MSCs of mesoderm or uncertain origin provided a supportive feeder layer for the self-renewal and colony-forming capacities of CD34^+^ HSPCs both in vitro and in vivo [14, 15]. However, the role of MSCs with neural crest origin derived from hPSCs on the maintenance of HSPCs has not been reported.

MSCs derived from dental pulp tissue (DP-MSC) differ from the other MSCs because their embryonic origin is the neural crest [16]. Mouse DP-MSCs have been reported to support in vitro growth of human UCB-HSPCs [17]. The successful differentiation of MSCs from human iPSCs via a neural crest cell lineage by traditional monolayer cultures has been reported [13]. However, it is a challenge to develop functional cells with full maturation from hPSCs. Recently developed 3D organoid methods may help to promote further diffusion of nutrient and differentiation factors through the scaffold to the embedded cells, thus aiding the development of fully functional mature cells [18]. The generation of functional NCCs has been reported and their properties have been well characterized using the 3D method in chicken, mice, and humans [19, 20]. 3D floating sphere cultures also maintain the neural crest-related properties of human dental pulp stem cells [21], and NCC-derived stem cells produced using 3D cultivation exhibit genetic stability and maintenance of stemness characteristics [22].

In the present study, we report a novel method for harvesting stromal cells from 3D human brain organoids under serum-free culture conditions. These long-term cultured organoid-derived stromal cells with neural crest cell lineages. They expressed high levels of MSC-specific markers; thus, we named pluripotent stem cell-derived MSCs (pMSCs). These pMSCs not only mimicked the morphology and function of UC-MSCs but also expressed additional functional molecules involved in regulation of the expansion and interaction of HSPCs. When UCB-CD34^+^ HSPCs were cocultured on UC-MSCs or pMSCs, the latter resulted in robust expansion of human UCB-CD34^+^ HSPCs in a long-term culture and efficient maintenance of their transplantability. Comparison by RNA sequencing indicated that coculture of human UCB-CD34^+^ HSPCs with pMSCs provided an improved microenvironment for HSC maintenance. The pMSCs highly expressed the Wnt signaling inhibitors SFRP1 and SFRP2, indicating that they may help to modulate the cell cycle to promote the expansion/maintenance of UCB-CD34^+^ HSPCs by antagonizing Wnt activation. Unlike traditional 2D culture methods, our method allowed all the multistep differentiation procedures to be completed in one vessel in 3D culture. Therefore, this represents a novel method for large-scale production of MSCs of neural crest origin and provides a potential approach for development of human hematopoietic stromal cell therapy for treatment of dyshematopoiesis.

## Material and methods

### Maintenance of hPSCs

The hiPSC lines were derived from human dermal fibroblasts (HDF) using exogenous factors (OCT4, SOX2, c-MYC, and KLF4) [23]. Briefly, 10,000 cells per plate from HDF at early passage were infected with pVSVG-based lentiviruses at a multiplicity of infection of 5 in 100 mm culture plates (Nunc, Denmark). After infection, the virus-containing solution was removed, and cells were cultured on irradiated mouse embryonic fibroblasts (MEF) in a humidified incubator at 37℃ and 5% CO2. The hiPSCs medium contained DMEM/F-12 (Life Technologies, USA), 20% knockout serum replacement (Life Technologies), 10 ng/mL human recombinant basic fibroblast growth factor (bFGF) (PeproTech), 0.1 mM β-mercaptoethanol (Life Technologies), 2 mM L-glutamine, 1% nonessential amino acids, and 1% penicillin/streptomycin (Life Technologies). Morphologically undifferentiated, colony-forming cells were picked and dissociated mechanically into small clumps with a micropipette tip 3–4 weeks after infection.

H1 hESCs were provided by Prof. Tao Cheng and maintained on Matrigel-coated plates in mTeSR1 medium (Stem Cell Technologies). The cells were dissociated into clumps using ReLeSR (Stem Cell Technologies) every 4–5 days.

### Culture of forebrain organoids from hPSCs

To generate forebrain-specific organoids with a modified step-wise protocol [24], hiPSC colonies were detached 7 days after passage with Collagenase Type IV, washed with fresh stem cell medium, and cultured in a 15 ml conical tube. On day 1, detached and washed hPSC colonies were transferred to an Ultra-Low attachment 6-well plate (Corning Costar), containing 3 ml of stem cell medium consisting of DMEM: F12, 20% KOSR, 1X Penicillin/Streptomycin, 1X Nonessential Amino Acids, 1X GlutaMAX, 2 µM Dorsomorphin (Sigma), and 2 µM A83-01 (MCE). On days 5–6, half of the medium was replaced with induction medium consisting of DMEM: F12, 1X N2 Supplement (Invitrogen), 10 µg/ml Heparin (Sigma), 1X Penicillin/Streptomycin, 1X Nonessential Amino Acids, 1X GlutaMAX, 1 µM CHIR99021 (MCE), and 1 µM SB-431542 (MCE). On day 7, organoids were embedded in Matrigel (Corning) and allowed to grow in induction medium for 6 more days. On day 14, embedded organoids were mechanically dissociated from Matrigel by pipetting up and down onto the plate with a 5 ml pipette tip. Typically, 10–20 organoids were transferred to each well of a 12-well spinning bioreactor containing differentiation medium, consisting of DMEM: F12, 1X N2 Supplements (Invitrogen), 1X B27 Supplements (Invitrogen), 1X Penicillin/Streptomycin, 1X Nonessential Amino Acids, and 2.5 µg/ml Insulin (Sigma).

### pMSC differentiation of hPSC-forebrain organoids

For MSC differentiation, brain organoids were adherently cultured in a petri dish at day 21. Differentiation medium was exchanged with serum-free MSC medium (MesenCult™-ACF Plus Medium; Stemcell Technologies) for 15 days; while, pMSCs were passaged from P0 to P3 [25]. The serum-free MSC medium is suitable for the isolation and expansion of tissue-derived MSCs. At confluence (85–90%), the cells were detached using trypsin/EDTA (0.05%; Thermo Fisher Scientific) and seeded at a density of 10,000 cells/cm^2^ in gelatin- (0.1%; Thermo Fisher Scientific) coated petri dish (Thermo). pMSCs were then cultured for 4–10 passages in serum-free MSC medium. The phenotype and multipotency of forebrain-specific organoid-derived MSCs (pMSC; including hiPSC-pMSCs, H1-pMSCs) were assessed by FACS analysis, and cells were tested for their ability to differentiate into mesenchymal-lineage cells (osteoblasts, adipocytes, and chondrocytes), as previously described [23].

### Isolation and culture of UC-MSCs

The use of the human umbilical cord tissue from a healthy donor, written informed consent was obtained from the pregnant women before labor. The approval of human umbilical cord-derived MSCs was obtained by the institutional ethics review committee of Institute of Blood Transfusion, Chinese Academy of Medical Sciences, China (approval number: 202030). The human UC-MSCs were manufactured as previously described [23]. Human umbilical cords were dissected longitudinally, and the arteries and veins were removed. The remaining pieces were chopped into 0.2 cm^3^ sections [23]. These explants were transferred in the CELLstart™ CTS™ Substrate- (Life Technologies, USA) coated 100 mm plates (Nunc, Denmark). StemPro1 MSC SFM XenoFree medium (Life Technologies, USA) with 1% penicillin streptomycin (Life Technologies, USA) was added to the plates, and the explants were cultured at 37 °C in a 5% CO2 incubator and left undisturbed to allow the cells to migrate from the explants. After 7–9 days, MSC-like cells were found around the fragments. The cells were passaged into another plate and further split 1:4 using 0.05% Trypsin–EDTA (Life Technologies, USA) once the cells reached 80% confluency.

### In vitro adipogenic, chondrogenic, and osteogenic differentiation

Induction of the differentiation of hiPSC-pMSCs was carried out for 21 days in different differentiation media [15]. A total of 10^4^ cells per well were seeded in six-well plates. To induce adipogenic differentiation, cells were cultured in an MSC medium supplemented with 50 μg/mL indomethacin (Sigma-Aldrich), 100 nM dexamethasone and 50 μg/mL ascorbic acid. For osteogenic induction, cells were cultured with MSC medium containing 0.5 μM ascorbic acid, 1 μM dexamethasone and 10 mM b-glycerol phosphate (all from Sigma-Aldrich). To induce chondrogenic differentiation, hiPSC-pMSCs were centrifuged in 0.2 mL of medium at 500 g for 10 min in 15 mL falcon tubes to form a pellet. The pellets were cultured in MSC medium supplemented with 397 μg/mL ascorbic acid-2-phosphate (Sigma-Aldrich), 1 mM sodium pyruvate (Sigma-Aldrich), 0.01 μM dexamethasone, 10 ng/mL TGF-β1 (Life Technologies), and 1% insulin-transferrin-selenium (Life Technologies). Adipogenesis was assessed by oil red staining, Osteogenesis by alizarin red staining, and chondrogenesis by alcian blue staining.

### Colony assay

The hematopoietic potential of cells was assessed by culture on methylcellulose (H4320, STEMCELL) supplemented with 1% penicillin/streptomycin (Invitrogen) and cytokines, as described previously [26]. BFU-E, CFU-Mix, CFU-GM, CFU-G, and CFU-M colonies were assessed after 10–16 days.

### Long-term culture initiating cell (LTC-IC) assays

The in vitro stroma supporting capacities of UC-MSCs and pMSCs were assessed using standard LTC-IC assays [27]. Briefly, 5 × 10^3^ MSCs were plated in gelatin-coated 96-well culture dishes and irradiated the following day (15 Gy). Primary human cord blood CD34^+^ cells were isolated from donor cord blood by magnetic activated cell sorting (Stem Cell). Twenty-four hours after irradiation, 1000 selected UCB-CD34^+^ cells were seeded and cultured in SFEM medium containing 50 ng/ml SCF, 30 ng/ml Flt3, and 30 ng/ml TPO with half medium changes every 3 days. After 6 weeks, cells were harvested by trypsinization and assayed for hematopoietic colony formation.

### HSCs transplantation

Mice were housed in the SPF-grade animal facility of the Center for Stem Cell Research and Application, Institute of Blood Transfusion. All animal experiments were approved by the Institutional Ethics Review Committee of Institute of Blood Transfusion (IERC-IBT). Adult BNDG mice were purchased from Beijing Biocytogen Co., Ltd. Human umbilical cord blood (UCB) nucleated cells containing 1 × 10^4^ CD34^+^ cells were cocultured with or without stromal cells for 2 weeks and transplanted through retro-orbital venous sinus into irradiated (1.0 Gy) 8–10-week-old BNDG mice [28]. Each mouse was anesthetized by the mixture of oxygen and isoflurane using the Matrx VMR Small Animal Anesthesia Machine, and the cells were injected into the retro-orbital vein of the irradiated recipients. The transplanted mice were maintained in Gentamicin sulfate-containing water for 2 weeks. Peripheral blood was obtained from the retro-orbital venous sinus for flow cytometric analysis 3 months post-transplantation. BM for flow cytometric analysis was obtained 6 months post-transplantation. The mice were euthanized with carbon dioxide. UCB nucleated cells were cultured in StemSpan™ SFEM II (Stemcell Technologies, Catalog No.: 09655) containing 50 ng/ml SCF, 10 ng/ml IL-11, 25 ng/ml Flt3, and 5 ng/ml TPO.

### Bulk cell RNA-seq

Total RNA was isolated from the H1 hESCs, hiPSCs, UC-MSCs, H1-pMSCs and hiPSC-pMSCs. UCB CD34^+^ cells after coculturing with and without MSCs for 7 days were sorted by magnetic beads (Stemcell Technologies, Catalog No.17856). Total RNA was isolated from the UCB CD34^+^ cells cocultured with and without UC-MSCs, H1-pMSCs and hiPSC-pMSCs, respectively. mRNA was purified from total RNA using poly-T oligo-attached magnetic beads. First strand cDNA was synthesized using random hexamer primer and M-MuLV Reverse Transcriptase. Second strand cDNA synthesis was subsequently performed using DNA Polymerase I and RNase H. The cDNA libraries were sequenced using an Illumina NovaSeq6000 system (Novogene Co., Ltd.). The cleaned FASTQ data were aligned to the HG38 human reference genome using HISAT2.

All RNA-Seq data can be found in the GEO database under accession code GSE220781. R packages of DESeq2 and clusterProfiler were used to statistically analyze RNA-seq data. Differential expression gene (DEG) of RNA-seq data was screened by DESeq2 with the threshold of *p*.adj < 0.05, and fold change > 1.5. GO enrichment analysis of indicated DEG list was performed by clusterProfiler.

### Statistical analysis

All data were statistically analyzed using the Graph Pad prism v 7.0 software. All data are presented as the mean ± SD. Statistical analyses were performed using the Student’s *t* test, NS: no significance, *P < 0.05, **P < 0.01.

## Results

### MSCs derived from human brain organoid cultures have MSC specific markers and neural properties

**Fig. 1:**
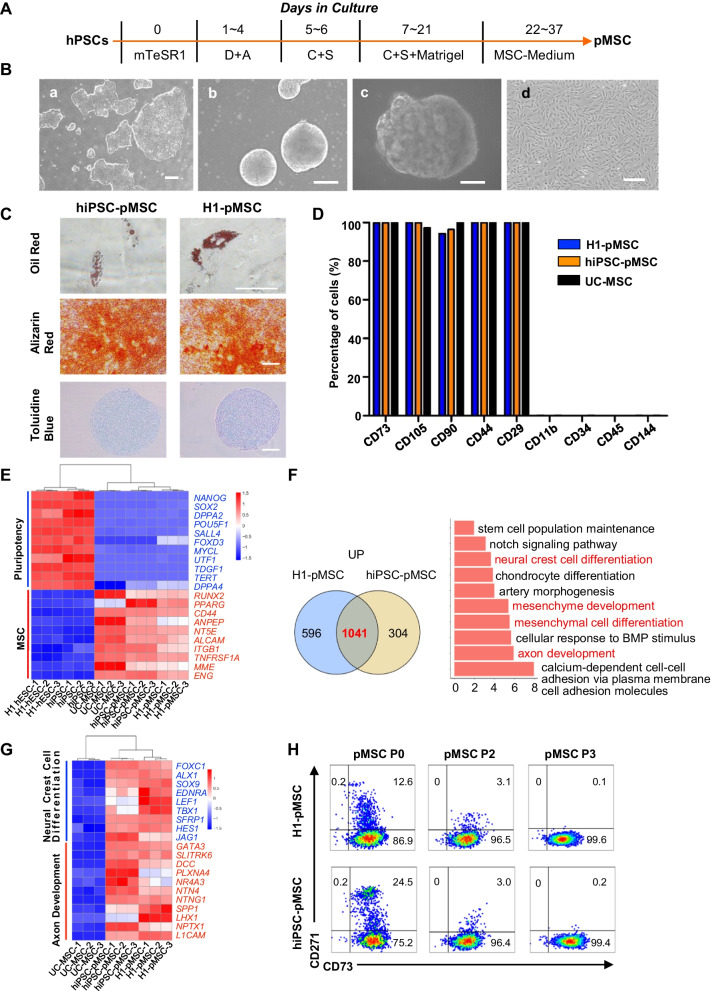
Human pluripotent stem cell-derived mesenchymal stem cells (pMSCs) reveal transcriptionally distinct populations. **A** Derivation of pMSCs from human brain-specific organoids. **B** Representative micrographs showing the typical cell morphology observed during the differentiation of hPSC to pMSCs. a: Day 0: hPSC colonies prior to differentiation; b: Differentiation of hESC into EB; c: human brain-specific organoids; d: pMSCs derived from hPSCs; scale bar, 200 µm. **C** Multilineage differentiation of pMSCs. After 2 weeks of corresponding induction, differentiated pMSCs were stained for mineralization with Alizarin Red, for chondrocytes with Toluidine Blue, or for lipid drops with Oil Red. **D** Percentage of surface markers in H1-MSCs, hiPSC-MSCs, and UC-MSCs. **E** Heatmaps showing genes related to pluripotency and MSC that were differentially expressed in H1-hESCs, hiPSCs, H1-MSCs, hiPSC-MSCs, and UC-MSCs. **F** Venn diagram and GO term analysis showing the number of common and distinct upregulated genes in the H1-MSCs and hiPSC-MSCs compared with UC-MSCs. **G** Heatmaps showing genes related to axon development and neural crest cell differentiation that were differentially expressed in H1-MSCs, hiPSC-MSCs, and UC-MSCs. **H** FACS analysis of surface markers of CD271 and CD73 in pMSCs from P0 to P3

Using a modified step-wise protocol of forebrain organoid cultures used in human pluripotent stem cells (hPSCs) lines [24], we derived human MSCs from both H1-hESC cells and human-induced pluripotent stem cell (hiPSC) forebrain organoids (Fig. 1A). Brain-specific organoids were generated from hPSCs at around day 21 (Fig. 1B). Cultures were digested with trypsin to generate single cell suspensions, and these cells were transferred to standard tissue culture plates in MSC medium. During repeated passaging, adherent cells gradually developed spindle-like morphology until a homogenous population developed over the course of several weeks (Additional file 1: Fig. S1A). The pMSCs were capable of differentiating into the classical mesenchymal-derived cells (osteoblasts, chondrocytes, and adipocytes) (Fig. 1C). Flow cytometric analysis indicated that, as for UC-MSCs, positive MSC markers were expressed by the vast majority of adherent MSC cells (> 99.8% for CD73, CD105, CD44, and CD29, and > 94.2% for CD90), whereas negative MSC markers, including CD11b, CD34, CD45, and CD144, were expressed by only a very small fraction of these cells (< 1%, Fig. 1D, Additional file 1: S1C). Both pMSCs and UC-MSCs grew rapidly at early passages, but their expansion potential slowed after several passages, almost plateauing at P14-P20 (Additional file 1: Fig. S1B). No significant difference in cell viability was observed between pMSCs and UC-MSCs (≥ 95.5%, Additional file 1: Fig. S1D).

In principal component analysis (PCA), pMSCs and UC-MSCs were separated from hPSCs according to PC1 and PC2. H1-pMSCs and hiPSC-pMSCs were positioned next to each other (Additional file 1: Fig. S1E). RNA-seq analysis indicated that there was a marked decrease in the expression of the pluripotent genes and an obvious increase in the expression of the MSC marker, adipogenic, and the osteochondrogenic progenitor genes in pMSCs and UC-MSCs, suggesting that pMSCs are MSC and tripotential in nature (Fig. 1E, Additional file 1: S1F). A total of 1041 genes were upregulated in either H1-pMSCs or hiPSC-pMSCs compared with UC-MSCs, and gene ontology analysis revealed that the upregulated genes were associated with axon development, mesenchyme development, and neural crest cell differentiation (Fig. 1F, G, Additional file 1: S1G). CD271, which is a neural crest marker, was also rapidly lost in response to exposure to the MSC media, and MSC markers such as CD73 also converged in passaged pMSCs to levels similar to those of UC-MSCs (Fig. 1H). The data indicated that we have developed an efficient and safe protocol to derive pMSCs from human brain organoid with neural crest origin.

### pMSCs support human UCB-HSPC expansion in vitro in a long-term culture

**Fig. 2:**
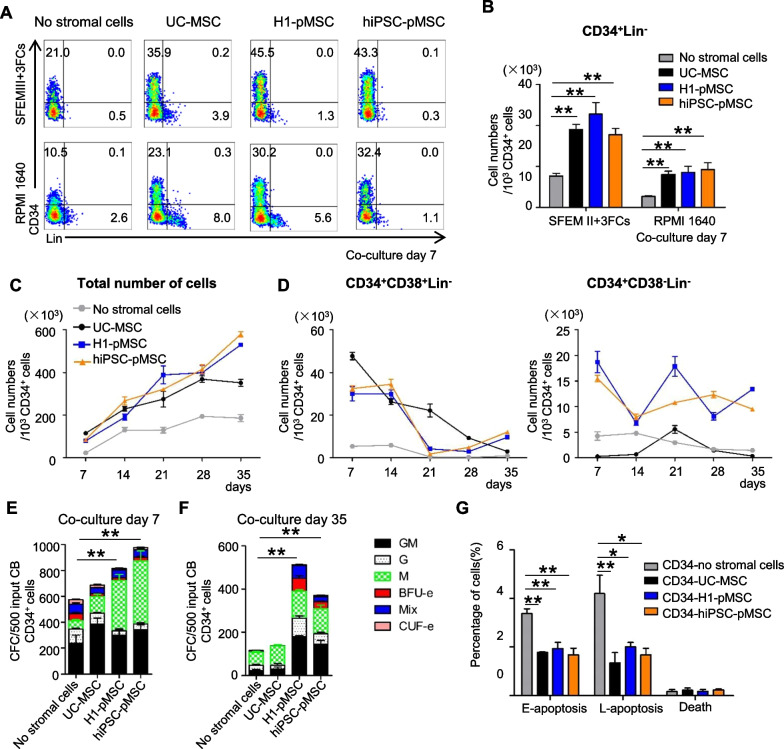
pMSCs support ex vivo maintenance and CFC expansion of UCB-CD34^+^ HSPCs. **A** Representative FACS analysis of UCB-CD34^+^ cells cocultured with and without H1-MSCs, hiPSC-MSCs, and UC-MSCs in 5% serum without added cytokines or SFEM medium with SCF, TPO, and FLT-3L for 7 days. **B** Cell yield of CD34^+^ Lin^−^ recovered from day 7 cocultures in 5% serum without added cytokines or SFEM medium with SCF, TPO, and FLT-3L. **C**, **D** Cell yield of total number of cells, CD34^+^CD38^+^ Lin^−^, CD34^+^CD38^−^Lin^−^ from days 7–35 after coculture with and without H1-MSCs, hiPSC-MSCs, and UC-MSCs, respectively. **E**, **F** CFC assay results of UCB CD34^+^ HSCs following 7 or 35 days coculture with and without H1-MSCs, hiPSC-MSCs, and UC-MSCs, respectively. Morphology of hematopoietic colonies after 14 days of culture in semisolid culture media based on methylcellulose at 37℃ and 5% CO2. **G** Quantification of apoptosis of hematopoietic cells on 4 culture conditions by AnnexinV/PI staining. The data in the bar graphs in panels **B**–**G** represent the mean ± SD. *N* = 3–4 replicates

To test for stroma function in vitro, the ability of pMSCs to support HSPCs was assessed by coculturing umbilical cord blood (UCB)-derived CD34^+^ cells (UCB-CD34^+^ cells) on monolayers generated from each mesenchymal population. FACS analysis and CFU assay were used to assay the effect of pMSCs on HSPCs (Additional file 1: Fig. S2A, B). Cultures were performed in basal medium with a low concentration of serum (5%) in α-MEM medium without added cytokines or SFEM medium with SCF, TPO and Flt-3L. Cocultures with pMSCs and UC-MSCs retained a significantly higher frequency and number of total CD34^+^Lin^−^ HSPCs than those without stromal cells in both mediums (Fig. 2A, B). When Lin^−^CD34^+^ CD38^−^ cells, a more primitive HSCs enriched population, were studied, the coculture system was modified by adding exogenous growth factors to allow sufficient cell growth for analysis. In the presence of cytokines, cocultures with pMSCs and UC-MSCs retained a significantly higher number of total cells than no stromal cells (Fig. 2C). Both mesenchymal populations from H1 hESCs and hiPSCs were able to support CD34^+^CD38^−^Lin^−^ cells for 1–5 weeks (Fig. 2D, Additional file 1: S2C). When more subpopulation of HSPCs was analyzed, the results indicated that higher proportion or absolute numbers of CD34^+^CD38^−^CD90^+^CD45RA^−^ long-term HSC (LT-HSCs), CD34^+^CD38^−^CD90^−^CD45RA^−^ short-term HSC (ST-HSC), CD34^+^CD38^−^CD90^−^CD45RA^+^ committed progenitors (C-progenitors) were observed in pMSCs cocultured group (*P* < 0.0001) (Additional file 1: Fig. S2D, E).

Given that UCB HSPC is multipotent and capable of differentiating into hematopoietic cell lineages with diverse biological functions, we examined the colony-forming cell potential of UCB CD34^+^ cells under four culture conditions using hematopoietic CFU assays. Significantly, more CFU colonies were maintained in pMSCs cocultures than UC-MSCs cocultures or no stromal cultures (Fig. 2E, Additional file 1: S2F). Standard LTC-IC assays were set up using irradiated pMSCs or UC-MSCs as feeder cells. After 5 weeks of coculture, hematopoietic colonies were generated from UCB-CD34^+^ cells on both feeder cell layers, with pMSCs showing a more potent in vitro stroma function than UC-MSCs (Fig. 2F), suggesting that pMSCs enhanced the overall differentiation potential of UCB-CD34^+^ cells and maintained their multipotency.

Given that pMSCs were associated with an increased proliferation rate of CD34^+^ cells, we further examined whether pMSCs inhibited apoptosis and thus increased the viability of CD34^+^ cells. We used Annexin V/7-AAD staining to identify apoptotic cells. The rates of early and late apoptosis were decreased in UCB-CD34^+^ cells cocultured with pMSCs and UC-MSCs (Fig. 2G). Thus, pMSCs and UC-MSC inhibit the apoptosis of UCB-CD34^+^ cells compared with those without stromal cells.

### pMSCs support transplantability of human UCB-HSCs

**Fig. 3:**
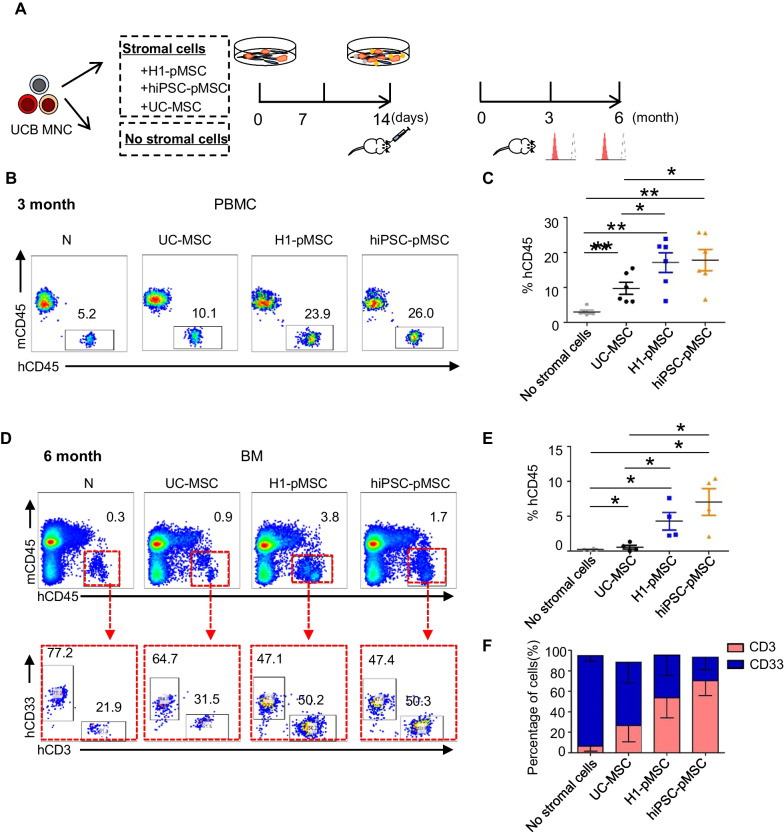
pMSCs improve human hematopoietic cell engraftment in NDG mice. **A** Scheme of the experiment design. Human cord mononuclear cells were cocultured with and without pMSCs and UC-MSCs for 2 weeks and transplanted into sublethally irradiated BNDG mice. Flow cytometry analysis was used to detect the chimerism of human blood cells in the recipient mice's peripheral blood or bone marrow at 3 or 6 months post-transplantation. **B** Representative flow plots of human cells (hCD45^+^) engraftment in PBMC of BNDG mice 3 months post-transplantation with human mononuclear cells cocultured with and without UCB-MSCs or pMSCs. **C** Engraftment (percent of human CD45^+^ cells) of mice 3 months after transplants. **D** Representative flow plots of human cells (hCD45^+^) engraftment in PBMC of BNDG mice 6 months post-transplantation with human mononuclear cells cocultured with and without UCB-MSCs and pMSCs. **E**, **F** Engraftment (percent of human CD45^+^ cells) and multilineage differentiation (CD33^+^ myeloid cells and CD3^+^T cells) of mice 6 months after transplants. The data in the bar graphs in panels **C**, **E**, **F** represent the mean ± SD. N = 4–6 replicates

To test for stroma function in vivo, a transplantation dose of total fresh cord blood nucleated cells, which contained 1 × 10^4^ CD34^+^ cells, was cocultured for 2 weeks with UC-MSCs, pMSCs, or without stromal cells, before total cells from the cocultures were transplanted into immunodeficient BNDG mice that had been subjected to sublethal doses of irradiation (Fig. 3A). Flow cytometry was used to analyze the chimerism of human blood cells in the peripheral blood or BM of recipient mice at 3 or 6 months post-transplantation. The results indicated that pMSCs cocultured cells produced significantly higher percentages of chimerism in mice peripheral blood than cells cultured with no stromal cells or with UC-MSCs at 3 months (Fig. 3B, C). The levels of engraftment in mice BM were also significantly higher in animals that received pMSCs cocultures compared with those that received no stromal or UC-MSCs cocultures at 6 months (Fig. 3D, E). Multilineage reconstitution (CD3^+^ lymphoid cells and CD33^+^ myeloid cells) was detected in the BM of all mice that received hematopoietic cells (Fig. 3F). Taken together, these data indicate that pMSCs are capable of maintaining engraftable HSPCs ex vivo.

### Coculture of HSPCs with pMSCs provides an improved microenvironment for HSC maintenance

**Fig. 4:**
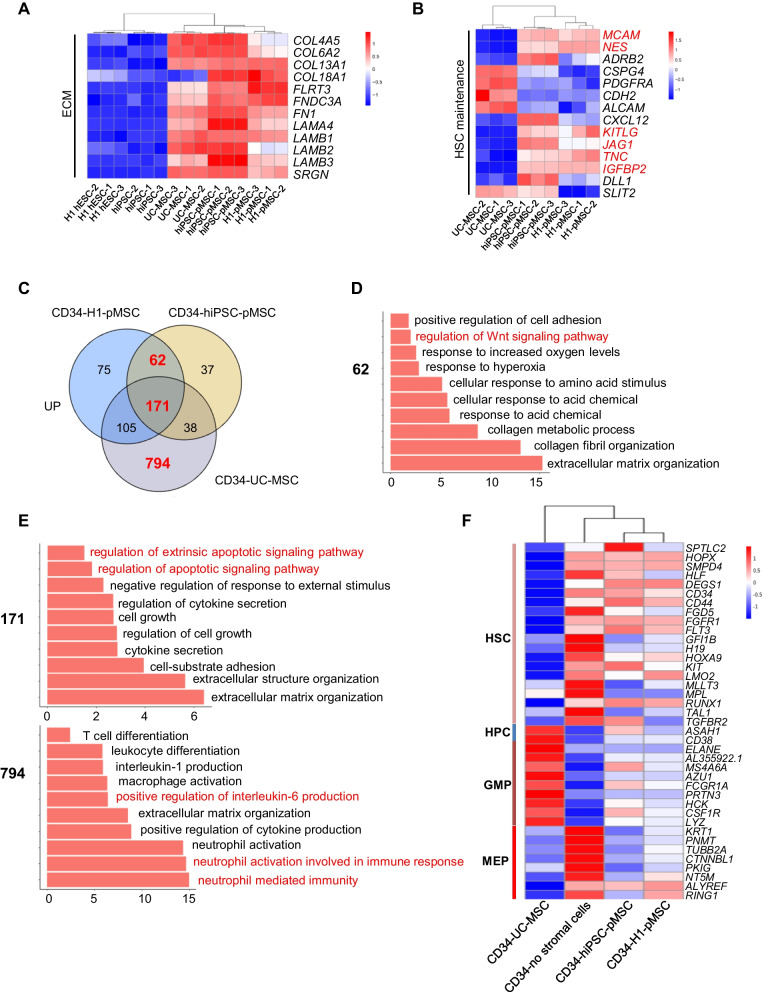
Transcriptome analysis for hematopoietic cells and MSCs. **A** Heatmaps showing genes related to ECM that were differentially expressed in H1-hESC, hiPSC, H1-MSCs, hiPSC-MSCs, and UC-MSCs. **B** Heatmaps showing genes related to HSC maintenance were differentially expressed in H1-MSCs, hiPSC-MSCs, and UC-MSCs. **C** Venn diagram showing the number of common and distinct upregulated genes in the CD34^+^ cells cocultured with H1-MSCs, hiPSC-MSCs, and UC-MSCs compared with that without stromal cells. **D**, **E** GO term analysis for common and distinct upregulated genes in the CD34^+^ cells cocultured with H1-MSCs, hiPSC-MSCs, and UC-MSCs compared with that without stromal cells. **F** HSC, HPC, GMP, and MEP signature gene expression in CD34^+^ cells cocultured with and without H1MSCs, hiPSC-MSCs, and UC-MSCs

To investigate the role of pMSCs in preserving HSPCs, we compared the transcriptome of pMSCs and UC-MSCs using RNA-seq analysis. Expression of extracellular matrix genes, included in collagen, fibronectin, laminin, and proteoglycans, was upregulated in both mesenchymal phenotypes (Fig. 4A), which is important given that the extracellular matrix (ECM) provides a structural scaffold that contributes to the maintenance of HSC homeostasis [29]. Enriched GO terms in upregulated genes in pMSCs included regulation of stem cell population maintenance, suggesting that pMSCs provide an advantage for HSPCs maintenance (Fig. 1F). Furthermore, pMSCs expressed significantly higher levels of *MCAM*(CD146); *NES* (NESTIN) [14, 30, 31]; and HSC regulatory genes, *KITLG*, *JAG1*, *IGFBP2*, and *TNC*, than UC-MSCs [32–35] (Fig. 4B). A total of 1047 genes were downregulated in either H1-pMSCs or hiPSC-pMSCs compared with UC-MSCs (Additional file 1: Fig. S3A). Enriched GO terms in downregulated genes in pMSCs included regulation of stem cell differentiation, IL-6 production, cytokine production, and neutrophil migration, further supporting the hypothesis that pMSCs provide an advantage in HSPCs maintenance (Additional file 1: Fig. S3B). Together, these findings suggest that pMSCs provide an improved microenvironment for HSC maintenance through a combination of mechanisms that involve both cell–cell contact and secreted factors.

To understand the molecular mechanisms governing how different niche cells contribute to the regulation of UCB-CD34^+^ cells, we performed RNA sequencing analysis on CD34^+^ cells at day 7 after coculturing with or without pMSCs or UC-MSCs. PCA and Pearson distance tree data showed that CD34^+^ cells cocultured with either H1-pMSCs or hiPSC-pMSCs shared a similar transcriptome profile to CD34^+^ cells cocultured with UC-MSCs or without stromal cells (Additional file 1: Fig. S3C). This suggests that H1-pMSCs and hiPSC-pMSCs have a similar effect on UCB CD34^+^ cells. A total of 62 genes were upregulated upon coculture of CD34^+^ cells with H1-pMSCs or hiPSC-pMSCs (Fig. 4C). Gene ontology analysis revealed upregulation of genes associated with extracellular matrix organization, collagen fibril organization, response to acid chemical and hyperoxia, and regulation of the Wnt signaling pathway (Fig. 4D), suggesting that coculture of UCB-CD34^+^ with pMSCs provides significant advantages in withstanding chemotherapy or extrinsic stimulation. A total of 171 genes were upregulated upon coculture of CD34^+^ cells with H1-pMSC, hiPSC-pMSCs, and UC-MSCs compared to culture without stromal cells. These genes are associated with extracellular matrix organization, extracellular structure organization, cell-substrate adhesion, cytokine secretion, cell growth, and regulation of cytokine secretion (Fig. 4E). A total of 794 genes were upregulated in CD34^+^ cells cocultured with UC-MSCs, with gene ontology analysis revealing upregulation of genes associated with neutrophil mediated immunity, neutrophil activation, and positive regulation of interleukin-6 production (Fig. 4E). Progenitor signature genes such as *CD38*, *CSF1R*, and *ELANE* were expressed at lower levels in CD34^+^ cells cocultured with pMSCs than in those cocultured with UC-MSCs [36]. Interestingly, coculture of CD34^+^ with pMSCs resulted in upregulated expression of *HOXA9*, *HLF*, and *KIT* HSC signature genes, consistent with their known functions associated with the primed state (Fig. 4F). Together, these findings suggest that UCB-CD34^+^ cells cocultured with pMSCs possess the HSC signature genes and the ability to withstand extrinsic stimulation.

### pMSCs express high levels of the Wnt inhibitors SFRP1, SFRP2, and IGFBP2

**Fig. 5:**
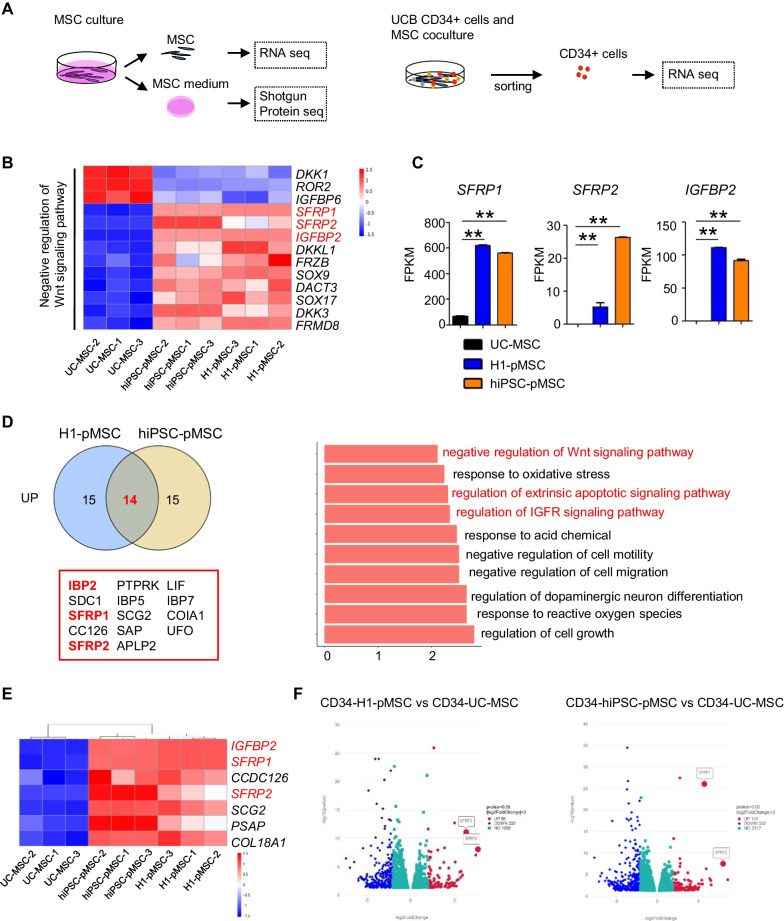
Wnt inhibitors of SFRP1 and SFRP2 are highly expressed in pMSCs and UCB-CD34^+^ cocultured with pMSCs. **A** Workflow of the experiment design with RNA and proteomic sequencing. Transcriptome of pMSCs and UC-MSCs were compared using RNA-seq analysis. Culture medium supernatant from pMSCs and UC-MSCs were compared by proteomic sequencing analysis. Transcriptome of UCB-CD34^+^ cells cocultured with and without MSCs for 7 days were compared using RNA-seq analysis. **B** Heatmaps showing genes related to negative regulation of Wnt signaling pathway were differentially expressed in H1-MSCs, hiPSC-MSCs, and UC-MSCs. **C** FPKM of *SFRP1*, *SFRP2*, and *IGFBP2* expressed in H1-MSCs, hiPSC-MSCs, and UC-MSCs. **D** Venn diagram and GO term analysis showing the number of common and distinct upregulated proteins in culture medium supernatant of H1-MSCs and hiPSC-MSCs compared with UC-MSCs. **E** Heatmaps showing genes coupregulated in H1-MSCs and hiPSC-MSCs both in RNA-seq and proteomics analysis. **F** Volcano Plot showing *SFRP1* and *SFRP2* upregulated in UCB CD34^+^ cells cocultured with H1-MSCs or hiPSC-MSCs compared that with UC-MSCs for 7 days. The data in the bar graphs in panels **B** represent the mean ± SD. *N* = 3–4 replicates

The MSCs and UCB-CD34^+^ cells cocultured on MSCs were analyzed by RNA-seq; while, the culture medium supernatant from pMSCs and UC-MSCs was analyzed by proteomic sequencing (Fig. 5A). Across the entire RNA-seq dataset, most of the genes involved in negative regulation of the Wnt signaling pathway were increased, including Secreted Frizzled-Related Protein 1 (*SFRP1)*, *SFRP2*, and *IGFBP2* (Fig. 5B). The gene whose expression was most altered between pMSCs and UC-MSCs was the Wnt antagonist *SFRP1* (Fig. 5C). Expression of other Wnt inhibitors was also significantly higher in pMSCs mesenchyme, including *SFRP2* and *IGFBP2* (Fig. 5C).

Proteomic sequencing analysis of culture medium supernatant from pMSCs and UC-MSCs revealed that a total of 14 proteins were upregulated in either culture supernatant of H1-pMSCs or hiPSC-pMSCs compared with UC-MSCs (Fig. 5D). Enriched GO terms for upregulated proteins in pMSCs included regulation of the insulin-like growth factor receptor signaling pathway and negative regulation of the Wnt signaling pathway (Fig. 5D). Seven proteins and genes were upregulated in either H1-pMSCs or hiPSC-pMSCs compared with UC-MSCs (Fig. 5E). Expression of both *SFRP1* (6.35 and 5.75-fold difference, *P* value < 0.01) and *SFRP2* (7.70 and 8.03-fold difference, *P* value < 0.01) was increased in UCB-CD34^+^ cocultured with pMSCs compared with UC-MSCs (Fig. 5F). Thus, pMSCs expressed higher levels of the Wnt inhibitors *SFRP1*, *SFRP2*, and *IGFBP2* than UC-MSCs.

### pMSCs may modulate cell cycle progression to promote transplantability of HSCs by antagonizing the Wnt signaling pathway

**Fig. 6:**
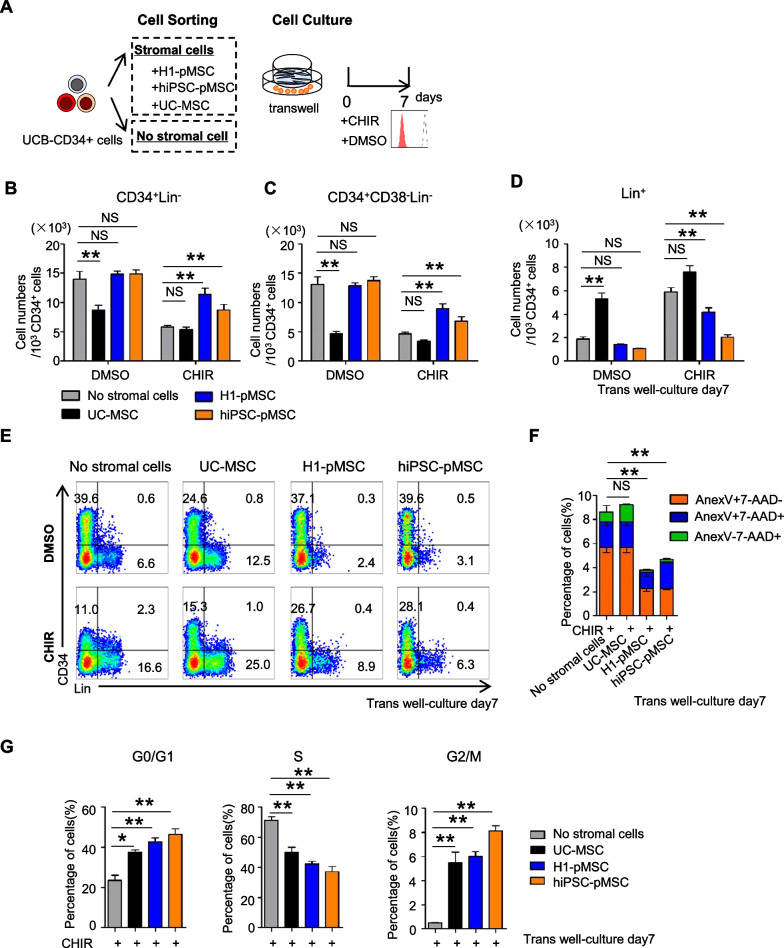
pMSCs antagonize Wnt signaling pathway activation and modulate the cell cycle of UCB-CD34^+^ cells. **A** Scheme of the experiment design. UCB-CD34 + cells transwell cocultured with and without pMSCs and UC-MSCs by addition of 3 µM CHIR99021 or DMSO. **B**–**D** CD34 + Lin-, CD34^+^ CD38^−^ Lin^−^, and Lin^+^ cell numbers after activation of Wnt signaling by addition of 3 µM CHIR99021 and DMSO. **E** Representative FACS analysis of CD34^+^ Lin^−^ cells at day 7 after coculture with and without H1-MSCs, hiPSC-MSCs, and UC-MSCs with addition of 3 µM CHIR99021 or DMSO. **F** The percentages of live, early apoptotic (E-apoptosis), late apoptotic (L-apoptosis), and dead cells in CD34^+^Lin^−^ cells at day 7 after coculture with and without H1-MSCs, hiPSC-MSCs, or UC-MSCs after activation of Wnt signaling by addition of 3 µM CHIR99021. **G** Representative flow cytometric analysis and quantification of BrdU incorporation and 7-AAD staining to determine the cell cycle distribution of 34^+^Lin^−^ HSPCs on day 7 of transwell culture after activation of Wnt signaling by addition of 3 µM CHIR99021. The data in the bar graphs in panels **B**–**G** represent the mean ± SD. *N* = 3–4 replicates

In order to fully understand the purpose of altered expression of Wnt inhibitors in pMSCs on UCB-CD34^+^ cells, we compared the effect of MSCs on CD34^+^ cells through transwell culture with the Wnt activator, CHIR99021 (CHIR), or DMSO (Fig. 6A). Addition of the Wnt agonist promoted rapid differentiation into Lin^+^ cells, and loss of CD34^+^Lin^−^ and CD34^+^CD38^−^Lin^−^ cells in both pMSCs and UC-MSCs cocultures (Fig. 6B–E). This latter observation is consistent with previous findings that canonical Wnt signaling regulates hematopoiesis in a dosage-dependent fashion, where high Wnt activation is detrimental to HSC self-renewal and leads to depletion of the LT-HSC pool [37, 38]. Interestingly, the proportion and number of CD34^+^Lin^−^ and CD34^+^CD38^−^Lin^−^ cells in pMSCs cocultures were still greater than those in UC-MSCs cocultures and no stromal cells with CHIR (Fig. 6B–E). This may be because Wnt inhibitors secreted from pMSCs resist the impact of CHIR on HSPC differentiation during transwell culture.

To determine whether pMSCs affect HSPC apoptosis and cell cycle, we performed flow cytometry to analyze apoptosis of CD34^+^Lin^−^ HSPC transwell cultures at day 7 with CHIR. The results showed that coculture of CD34^+^Lin^−^ cells with pMSCs significantly reduced the percentage of early apoptotic cells and significantly increased the percentage of live cells (Fig. 6F). 5′-Bromo-2′-deoxyuridine (BrdU) incorporation analysis indicated that the cell cycle of 34^+^Lin^−^ cells differed between pMSCs and UC-MSCs cocultures. The decreased differentiation of CD34^+^Lin^−^ cells upon pMSCs treatment was due to the induction of quiescence; while, CHIR promoted the progression of 34^+^Lin^−^ cells without coculture into S phase (Fig. 6G).

Taken collectively, the entirety of the data indicates that pMSCs exhibit elevated expression levels of Wnt inhibitors, notably including *SFRP1*, *SFRP2*, and *IGFBP2*. pMSCs inhibited apoptosis and increased quiescence of HSPC when exposed to CHIR. pMSCs may exert their effects through suppression of Wnt signal and modulation of the cell cycle to maintain human transplantable HSCs. These findings suggest that UCB-CD34^+^ cells cocultured with pMSCs possess the required self-renewal potential to provide long-term multipotent reconstitution and withstand extrinsic stimulation, partially by inhibiting the Wnt signaling pathway.

## Discussion

In this study, we have described a detailed protocol to derive pMSCs from 3D human brain organoid culture. These pMSCs were most likely developed from the NCC lineage, and they supported the expansion and maintenance of transplantability of human UCB-CD34^+^-HSPCs when cocultured under long-term, serum-free conditions. RNA sequencing analysis indicated that coculture with pMSCs provided an improved microenvironment for functional maintenance of human HSCs via a comprehensive mechanism involving cell–cell contact and the secretion of specific factors. The pMSCs may also modulate the cell cycle to exert their effects on promoting the expansion and maintenance of UCB-CD34^+^-HSPCs through antagonizing Wnt signal activation.

It has been demonstrated that during embryonic development, MSCs arise from two major sources: neural crest and mesoderm [39, 40]. Based on our observations that the pMSCs were generated along with the development of an NCC lineage and exhibited typical MSC characteristics similar to those of UC-MSCs, as indicated by the expression of surface markers, proliferation patterns, and differentiation function. Notably, pMSCs exhibited several neural cell properties, as evidenced by their elevated expression of genes related to axon development, chondrocyte and NCC differentiation. CD271 was reported as a marker for NCC [13]. During the second passage of MSC culture from separated brain organoid cells, the proportion of NCCs (CD271 + cells) drastically increased (from 12.6 to 24.5% of total cells) and then gradually faded along with the development of the purified pMSCs population. Given that no cell components of mesodermal cell lineage were observed in the brain organoid after 21 day culture (data not shown), the separated cells that subsequently developed into MSCs were unlikely to have derived directly from mesodermal precursors. This strongly suggests that our pMSCs originate from the NCC lineage.

Similarly, recent reports have shown that purified hPSC-NCC precursor could give rise to large production of MSCs, which play a role as regenerative substitutes, when applied to model mice [13]. However, in these reports, MSCs were all produced in 2D cultures and required several purification and manipulation steps, thus hampering mass production. Traditional monolayer cultures allow the external control of targeted hiPSC differentiation to produce more uniform cell populations [12, 41–43]. However, these cultures lack the 3D cell assembly properties that could define endogenous biological systems. It was recently reported that MSC-derived conditioned medium (MSC-CM) produced mainly from 3D cultures has the potential to increase hepatic stem/progenitor cell differentiation compared to the medium produced from 2D cultures, suggesting that 3D culture-derived MSCs produce soluble factors that are critical to these processes [44]. Recent organoid technology has allowed the development of optimized 3D aggregates of stem cells, which are able to self-organize and develop disparate tissues in vitro, similar to teratoma formation in vivo [24]. Through RNA sequencing analysis, we have clearly demonstrated that the 3D brain organoid-derived pMSCs possess many properties of functional neural cells and were largely different from UC-MSCs. Thus, in our culture system, the long-term 3D brain organoid culture-derived NCC precursors might also generate pMSCs with more functional maturity.

Although many reports have shown that scalable production of MSCs can be achieved from hPSCs [11–13, 43, 45], the heterogeneous origin of hPSC-derived MSCs means they are likely to have different functions, which would limit their clinical applications. When generated and selected in MSC inducing media, hPSC-derived MSCs with mesoderm or uncertain origin coexpress CD105 and CD73, and can functionally provide a supportive feeder layer for CD34^+^ HSC self-renewal and colony-forming capacities in vitro and in vivo, similar to BM-MSCs [14, 15]. The developing mouse BM harbors different MSC populations with distinct origins and specialized roles. In the mouse BM, nestin^−^ MSCs derived from the mesoderm have a primarily osteochondral progenitor function. In contrast, a distinct population of neural crest-derived nestin^+^ MSCs contributes to directed HSC migration through the secretion of the chemokine Cxcl12 to ultimately establish the HSC niche in the neonatal BM [46]. However, the role of maintenance on human CD34^+^ HSPCs by hPSC-derived MSCs with NCC origin has not been reported. In the present study, we have compared the reconstitution ability of UCB-CD34^+^ HSCs in two cocultures, either with pMSCs or UC-MSCs in an LTC-IC assay. Our results clearly demonstrate that coculture with pMSCs greatly supports the expansion and maintenance of the function of UCB-CD34^+^ HSCs, both in vitro and in vivo.

MSCs play an important role in modulating the BM microenvironment and supporting hematopoiesis [47]. The niches of HSCs can be viewed as a network consisting of niche cell types, membrane-bound or secreted signaling molecules of the cytokine or chemokine families and a complex extracellular matrix (ECM) [48]. The ECM provides a structural scaffold that contributes to the maintenance of HSC homeostasis [29, 49]. A report demonstrated that the nestin^+^ mesenchyme in the murine fetal liver HSC niche expresses *MCAM*(CD146) [50], further supporting the inclusion of *MCAM* (CD146) as a defining factor for mesenchyme with HSC niche activity. It was also reported that CD146^+^ perivascular cells support the human adult hematopoietic stem cells in the BM [51]. In the present study, both H1-pMSCs and hiPSC-pMSCs exhibited upregulated expression of extracellular matrix genes, including collagen, fibronectin, laminin, and proteoglycans, while also expressing known HSC niche genes such as *NES* and *MCAM*, indicating that direct contact of HSCs with pMSCs might contribute to their improved function. Different reports have highlighted that soluble factors released by MSCs are sufficient to promote the expansion of hematopoietic cells [52, 53]. For example, human IGFBP2 is a secreted protein that helps to enhance ex vivo expansion of HSCs [32]. Here, the coexpression of hematopoietic niche factors, such as *KITL*, *JAG1*, and *IGFBP2*, at a specifically high level provides further evidence that pMSCs play a unique role in supporting HSC functions. We also detected higher expression of IGFBP2 protein in the medium of pMSC through proteomic sequencing analysis. Since these components of the hematopoietic niche play a critical role in the maintenance of HSC function in vivo, we conclude that a subset of our pMSCs supported HSPCs through a comprehensive mechanism that might involve cell–cell contact and the secretion of specific factors. The prospect of isolating human stroma with the capacity to support HSCs is particularly exciting given the strong clinical interest in using stroma coculture as a platform for HSC expansion. Thus, our study highlights a possible role for pMSCs in HSC-based clinical applications.

The members of the Wnt signaling pathway have emerged as critical regulators of HSC self-renewal and proliferation [54]. Moreover, inhibition of Wnt signaling in murine BM-MSCs cocultures significantly enhances LT-HSC expansion [55]. Both SFRP1 and SFRP2 are known as important modulators of Wnt signaling, which bind to Wnt ligands directly [56]. *SFRP1* and *SFRP2* are expressed in BM osteoblasts and differentially regulate HSC functions in mice [57]. Previous studies have demonstrated that upregulation of SFRP1 or SFRP2 in mouse embryo-derived stromal cell lines enhances their supportive effect on cultured HSCs [5, 58]. Serial BMT assays demonstrated that the maintenance of self-renewing HSCs is negatively affected in the *sfrp1*−/− bone microenvironment [59]. Treatment of HSPCs with exogenous SFRP1 protein before transplantation damages HSC engraftment efficiency [57]. In contrast to the results with SFRP-1, LT-HSCs treated with SFRP-2 ex vivo led to an increase in HSC engraftment capacity [57]. Based on the published literature, secreted SFRP2 from mineralized osteoblasts could inhibit hematopoietic differentiation by increasing the proliferation of self-renewing HSCs [57].Thus, the SFRPs produced by mouse BM stromal cells and embryo-derived stromal cell lines may well influence the behavior of their neighboring hematopoietic cells but in a somewhat comprehensive and reciprocal manner. The role of SFRP1 and SFRP2 in human MSCs on HSPCs maintenance has not been reported. In the current study, we discovered that the expression of SFRP1 and SFRP2 was overwhelmingly higher in the pMSCs than MSCs from other sources, as confirmed by RNA sequencing and proteomic sequencing. Therefore, pMSCs might exert their effects through suppression of Wnt signaling and modulation of the cell cycle to maintain the function of HSCs.

In summary, we have described a novel method to produce brain organoid-derived pMSCs generated from hPSCs. The pMSCs produced in our 3D serum-free culture system have several advantages over those produced in 2D systems. Firstly, a long-term 3D organoid culture ensured a functionally mature source of MSC precursor cells (probably derived from NCCs) that could derive pMSCs with functional maturities. Secondly, the neural origin of pMSC avoided development from other mesodermal precursors to form a mass mixture of mesoderm differentiation, which is dominantly conducted during the MSC cultures from hPSC-derived immature cells. This ensured a comparatively pure population of pMSCs with highly neural properties. Thirdly, the unique function of pMSCs to support the expansion and maintenance of UCB-CD34^+^ HSPCs, especially by enhancing the function of transplantable HSCs, suggested they have great clinical potential. There have been few reports of homologous HSC-supporting stromal cell lines as the universal therapeutic product; however, our system to culture pMSCs has the potential to be easily scaled and translated into a system for drug development. Finally, the pMSCs made from individual hiPSCs would provide the ideal model for the study of the effect of neural microenvironments on hematopoietic regulation in normal or disease states.

## Conclusions

A novel method for harvesting MSCs with neural crest origin from 3D human brain organoids under serum-free culture conditions was reported. The pMSCs not only mimicked the morphology and function of umbilical cord MSCs (UC-MSCs) but also expressed additional functional molecules involved in maintenance of UCB-HSPC. RNA and proteomic sequencing indicated that pMSCs provided an improved microenvironment for HSC maintenance via mechanisms involving cell–cell contact and secreted factors. The pMSCs highly expressed the Wnt signaling inhibitors SFRP1 and SFRP2, indicating that they may help to modulate the cell cycle to promote the maintenance of UCB-CD34^+^ HSPCs by antagonizing Wnt activation. This represents a novel method for large-scale production of MSCs of neural crest origin and provides a potential approach for development of human hematopoietic stromal cell therapy for treatment of dyshematopoiesis.

### Supplementary Information

**Additional file 1:** Mesenchymal stem/stromal cells from human pluripotent stem cell-derived brain organoid enhance the ex vivo expansion and maintenance of hematopoietic stem/progenitor cells.

## Data Availability

The data discussed in this publication have been deposited in NCBI’s Gene Expression Omnibus and are accessible through GEO Series accession number GSE220781.

## Abbreviations

AGM: Aorta-gonad-mesonephros
bFGF: Basic fibroblast growth factor
BM: Bone marrow
BM-MSCs: Bone marrow-derived MSCs
C-progenitors: Committed progenitors
DP-MSC: Dental pulp tissue-derived MSCs
ECM: Extracellular matrix
HSPCs: Hematopoietic stem/progenitor cells
HSCs: Hematopoietic stem cells
hPSCs: Human pluripotent stem cells
hiPSC: Human induced pluripotent stem cell
HDF: Human dermal fibroblasts
HBSS: Hanks’ balanced salt solution
LT-HSCs: Long-term HSC
LTC-IC: Long-term culture initiating cell
MEF: Mouse embryonic fibroblasts
MSCs: Mesenchymal stem/stromal cells
MSC-CM: MSC-derived conditioned medium
NCCs: Neural crest cells
pMSCs: Pluripotent stem cell-derived MSCs
PCA: Principal component analysis
SFRP1: Secreted frizzled-related protein 1
UC-MSCs: Umbilical cord-derived MSCs
UCB: Umbilical cord blood
