# Supplementary material for: Mesenchymal stem/stromal cells from human pluripotent stem cell-derived brain organoid enhance the ex vivo expansion and maintenance of hematopoietic stem/progenitor cells
Source: Stem Cell Res Ther. 2024 Mar 5;15:68. doi: 10.1186/s13287-023-03624-w (PMC10916050; doi:10.1186/s13287-023-03624-w)
Supplement: Supplementary file 1 — Additional file 1: Mesenchymal stem/stromal cells from human pluripotent stem cell-derived brain organoid enhance the ex vivo expansion and maintenance of hematopoietic stem/progenitor cells. [file 13287_2023_3624_MOESM1_ESM.pdf]

Supplemental Information

Mesenchymal stem/stromal cells from human pluripotent stem cell-derived brain organoid enhance the ex vivo expansion and maintenance of hematopoietic stem/progenitor cells

Ya Zhou[1#], Xinping Cai[1,2#], Xiuxiu Zhang[1], Yong Dong[1,3], Xu Pan[1], Mowen Lai[1], Yimeng Zhang[1], Yijin Chen[1], Xiaohong Li[1], Xia Li[1], Jiaxin Liu[1], Yonggang Zhang[1,*], Feng Ma[1,*]

Supplementary Information

Zhou et al., Mesenchymal stem/stromal cells from human pluripotent stem cell-derived brain organoid enhance the ex vivo expansion and maintenance of hematopoietic stem/progenitor cells

Supplemental Experimental Procedures

Flow cytometry and cell sorting

Cocultured cells were dissociated with 0.25% trypsin-EDTA solution (Invitrogen) and filtered through a 70 μm nylon mesh to obtain a single-cell suspension. Flow cytometry was performed using a FACSCanto II system (BD Biosciences), and data were analyzed using FlowJo software (v10.0.8.). Cell sorting was performed using a MoFlo Astrios High Speed Cell Sorter (Beckman Coulter). The antibodies used are presented in Table S1.

Cell cycle analysis

Cells were treated with BrdU for 6 h, stained for surface antigens, and processed using an APC-BrdU Flow Kit (BD) according to the manufacturer's instructions.

Apoptosis assays

Apoptotic cells were stained with annexin V–APC and 7-AAD according to the manufacturer's instructions (BioLegend, Catalog No.:640930). Cell apoptosis was detected by using a FACSCanto II system (BD Biosciences).

Supplemental Figures and Legends

Supplemental table S1. Antibodies used for flow cytometric analysis

**Antigen**	**Fluor chrome Conjugated**	**Source**	**Clone**	**Isotype**	**Cat. No.**
CD271	PE-CY7	BD	C40-1457	Mouse IgG1, κ	562122
CD73	APC	BD	TY/11.8	Mouse IgG3, κ	560847
CD73	PE	Biolegend	AD2	Mouse IgG1, κ	344004
CD105	PE	BD	266	Mouse IgG1, κ	560839
CD90	APC-CY7	Biolegend	5E10	Mouse IgG1, κ	328132
CD44	FITC	BD	G44-26	Mouse IgG2b, κ	555478
CD29	PE	Biolegend	TS2/16	Mouse IgG1, κ	303004
CD11b	APC	Biolegend	M1/70	Rat IgG2b, κ	101212
CD34	APC	BD	581	Mouse IgG1, κ	555824
CD45	APC-CY7	Biolegend	2D1	Mouse IgG1, κ	368516
CD45	FITC	Biolegend	30-F11	Rat IgG2b, κ	103108
CD144	Alexa Fluor 647	BD	55-7H1	Mouse IgG1, κ	561567
Lineage Cocktail (CD3, CD14, CD16, CD19, CD20, CD56)	FITC	Biolegend	UCHT1; HCD14; 3G8; HIB19; 2H7; HCD56;	Mouse IgG1, κ	348801
CD38	PE	BD	HIT2	Mouse IgG1, κ	555460
CD45RA	PE-CY7	Biolegend	HI100	Mouse IgG2b, κ	304126
CD33	PE-CY7	Biolegend	P67.6	Mouse IgG1, κ	366617
CD3	PE	Biolegend	UCHT1	Mouse IgG1, κ	980008
7-AAD		BD			559925

Supplemental Figure Legends

Figure S1. Characterization of P-MSCs, related to Figure 1

(A) Schematic diagram and representative micrographs showing the typical cell morphology of H1-MSCs, hiPSC-MSCs, and UC-MSCs; scale bar, 200 μm.

(B) Growth curves of UC-MSCs and P-MSCs (n = 3).

(C) FACS analysis of surface markers in H1-MSCs, hiPSC-MSCs, and UC-MSCs.

(D) Histograms showing the viability (7-AAD-AnnexV-) of H1-MSCs, hiPSC-MSCs, and UC-MSCs.

(E) Principal-component analysis plots of H1-hESCs, hiPSCs, H1-MSCs, hiPSC-MSCs, and UC-MSCs.

(F) Heatmaps showing adipogenic and osteochondrogenic progenitor genes that were differentially expressed in H1-hESCs, hiPSCs, H1-MSCs, hiPSC-MSCs, and UC-MSCs.

(G) Heatmaps showing genes related to mesenchyme development that were differentially expressed in H1-MSCs, hiPSC-MSCs, and UC-MSCs.

Figure S2. Analyses of alternative mesenchymal and hematopoietic populations, related to Figure 2

(A) Scheme of the experiment design. Fresh $CD34^+$ HSPCs in human cord mononuclear cells were sorted by CD34 magnetic beads.

(B) Representative FACS analysis of $CD34^+$ cells in human cord mononuclear cells sorted by CD34 magnetic beads.

(C) Percentage of $CD34^+CD38^+$ Lin^- and $CD34^+CD38^-$ Lin^- cells from day 7 to 35 after coculture with and without H1-MSCs, hiPSC-MSCs, or UC-MSCs.

(D, E) Representative FACS analysis and cell yield of $CD34^+$ $CD38^-$ $CD90^+$ $CD45RA^-$ LT-HSCs, $CD34^+$ $CD38^-$ $CD90^-$ $CD45RA^-$ ST-HSC, $CD34^+$ $CD38^-$ $CD90^-CD45RA^+$ committed progenitors (C-progenitors), and $CD34^+$ $CD38^+$ progenitors at day 7 after coculture with and without H1-MSCs, hiPSC-MSCs, or UC-MSCs.

(F) Representative micrographs and May-Grunwald-Giemsa staining of CFU erythrocyte (CFU-E), Burst-forming-unit erythrocyte (BFU-E), CFU granulocyte/erythrocyte/monocyte/megakaryocyte (CFU-GEMM), CFU monocyte (CFU-M), CFU granulocyte (CFU-G), and CFU granulocyte/monocyte (CFU-GM); scale bar, 50 μm.

Figure S3. Transcriptome analysis for MSCs and UCB-$CD34^+$ cells cocultured with and without MSCs, related to Figure 4

(A, B) Venn diagram and GO term analysis showing the number of common and distinct downregulated genes in the H1-MSCs and hiPSC-MSCs compared with UC-MSCs.

(C) PCA analysis and Pearson distance tree for $CD34^+$ HSPCs cocultured with and without H1-MSCs, hiPSC-MSCs, and UC-MSCs.

Figure S1.

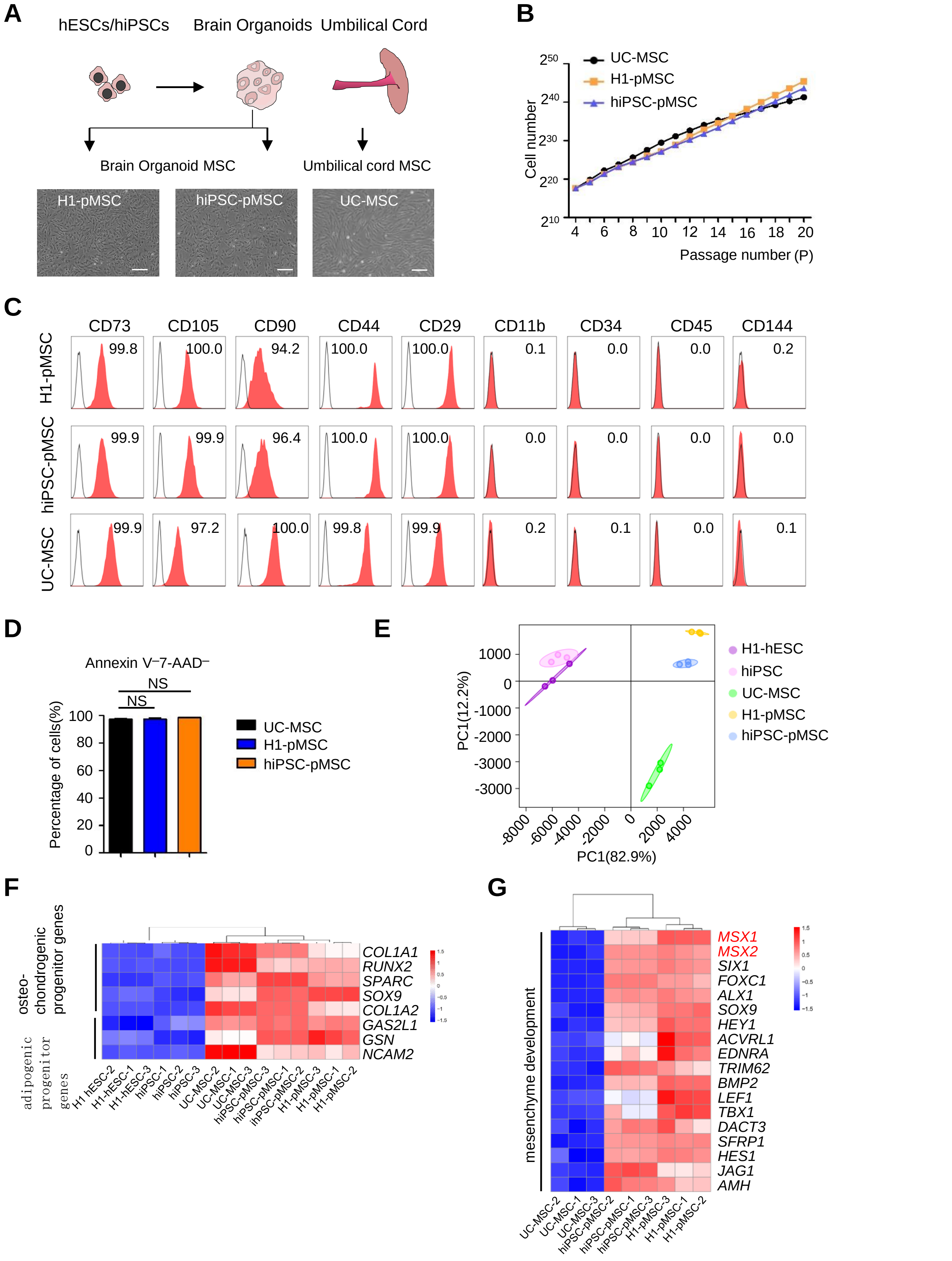

A
hESCs/hiPSCs
Brain Organoids
Umbilical Cord
Brain Organoid MSC
Umbilical cord MSC
H1-pMSC
hiPSC-pMSC
UC-MSC
B
UC-MSC
H1-pMSC
hiPSC-pMSC
Cell number
Passage number (P)
C
CD73
CD105
CD90
CD44
CD29
CD11b
CD34
CD45
CD144
H1-pMSC
hiPSC-pMSC
UC-MSC
D
Annexin V⁻7-AAD⁻
NS
NS
Percentage of cells(%)
UC-MSC
H1-pMSC
hiPSC-pMSC
E
H1-hESC
hiPSC
UC-MSC
H1-pMSC
hiPSC-pMSC
PC1(12.2%)
PC1(82.9%)
F
osteo-chondrogenic progenitor genes
adipogenic progenitor genes
COL1A1
RUNX2
SPARC
SOX9
COL1A2
GAS2L1
GSN
NCAM2
G
mesenchyme development
MSX1
MSX2
SIX1
FOXC1
ALX1
SOX9
HEY1
ACVRL1
EDNRA
TRIM62
BMP2
LEF1
TBX1
DACT3
SFRP1
HES1
JAG1
AMH

Figure S2.

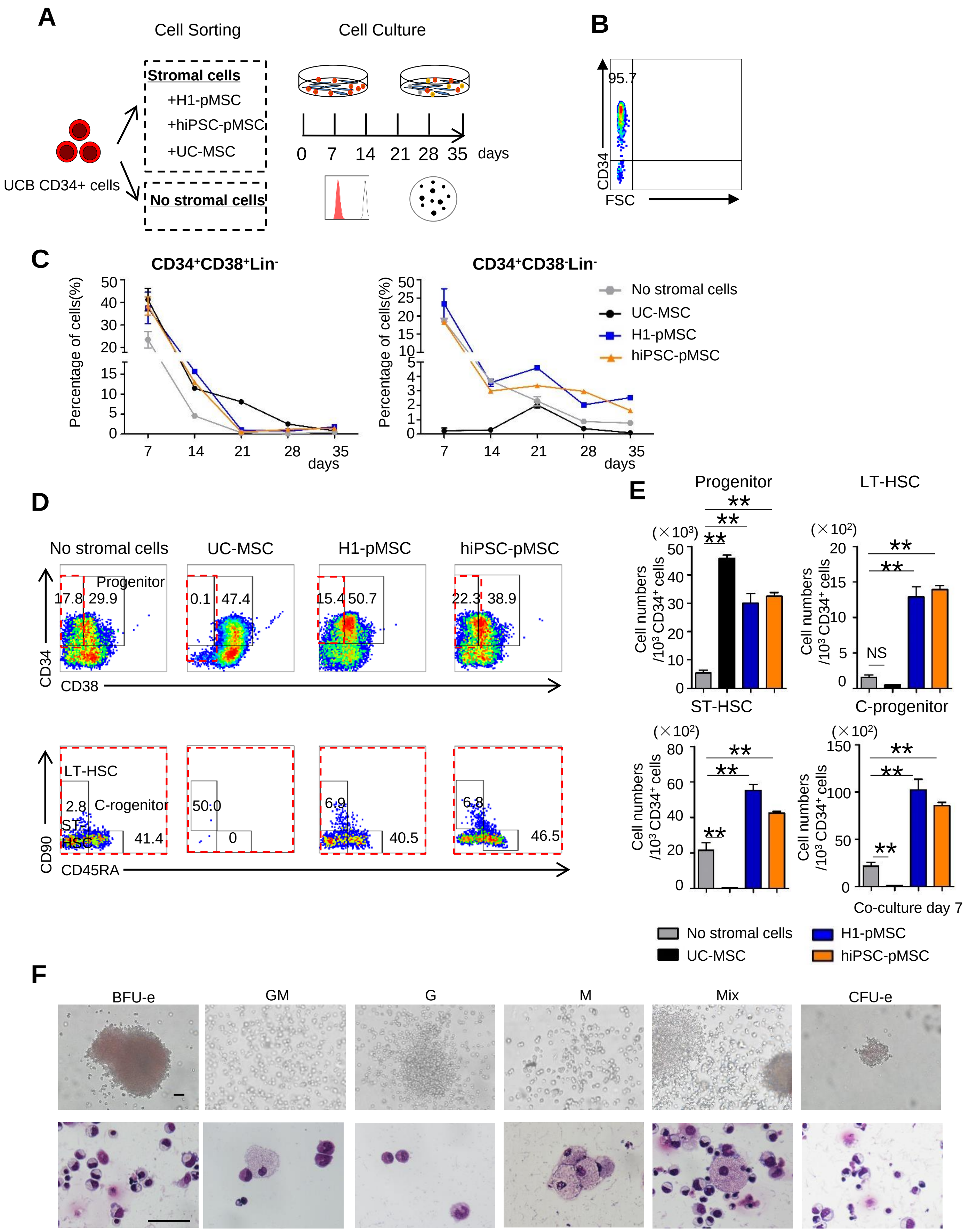

A
Cell Sorting
Cell Culture
Stromal cells
+H1-pMSC
+hiPSC-pMSC
+UC-MSC
No stromal cells
UCB CD34+ cells
0 7 14 21 28 35 days
B
95.7
CD34
FSC
C
CD34+CD38+Lin-
CD34+CD38-Lin-
Percentage of cells(%)
days
No stromal cells
UC-MSC
H1-pMSC
hiPSC-pMSC
D
No stromal cells
UC-MSC
H1-pMSC
hiPSC-pMSC
Progenitor
17.8 29.9
0.1 47.4
15.4 50.7
22.3 38.9
CD34
CD38
LT-HSC
2.8 C-rogenitor
ST-HSC
41.4
50.0
0
6.9
40.5
6.8
46.5
CD90
CD45RA
E
Progenitor
LT-HSC
Cell numbers /10³ CD34+ cells
ST-HSC
C-progenitor
NS
Co-culture day 7
No stromal cells
UC-MSC
H1-pMSC
hiPSC-pMSC
F
BFU-e
GM
G
M
Mix
CFU-e

Figure S3.

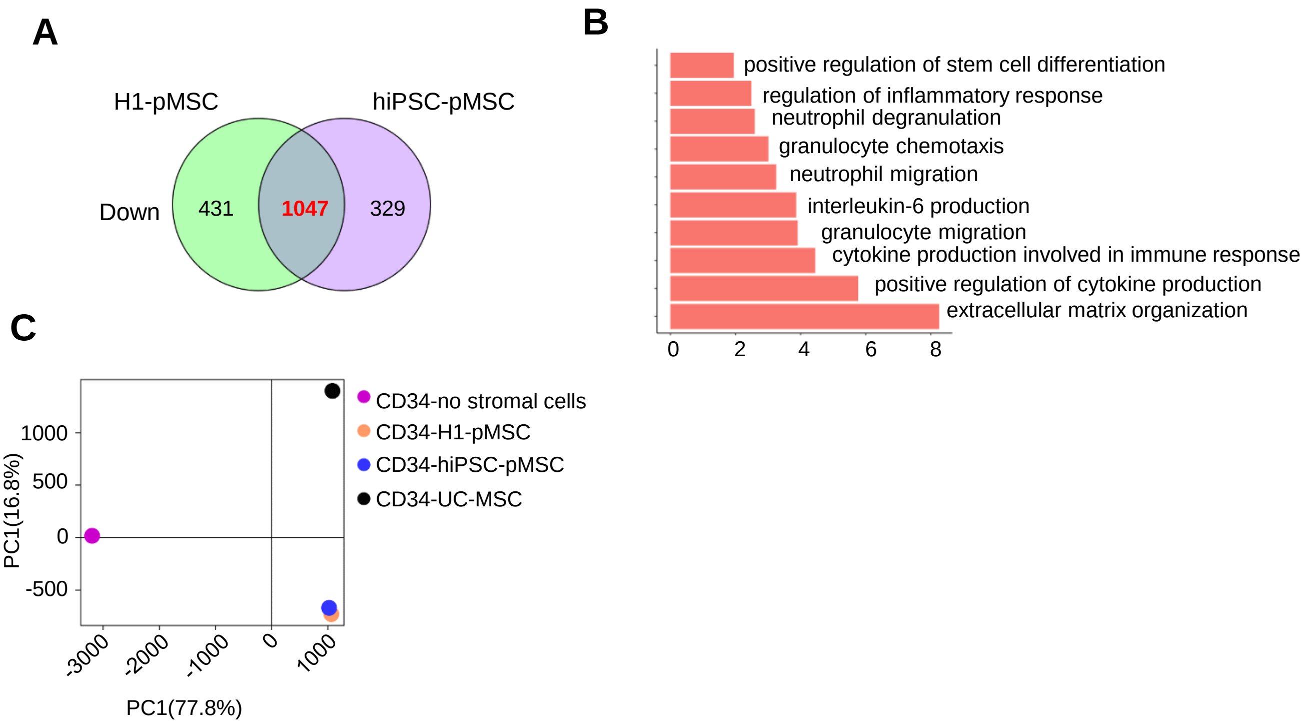
A
H1-pMSC
hiPSC-pMSC
Down
431
1047
329
B
positive regulation of stem cell differentiation
regulation of inflammatory response
neutrophil degranulation
granulocyte chemotaxis
neutrophil migration
interleukin-6 production
granulocyte migration
cytokine production involved in immune response
positive regulation of cytokine production
extracellular matrix organization
0
2
4
6
8
C
CD34-no stromal cells
CD34-H1-pMSC
CD34-hiPSC-pMSC
CD34-UC-MSC
1000
500
0
-500
PC1(16.8%)
-3000
-2000
-1000
0
1000
PC1(77.8%)
